# DNA Repair Biomarker for Lung Cancer Risk and its Correlation With Airway Cells Gene Expression

**DOI:** 10.1093/jncics/pkz067

**Published:** 2019-09-12

**Authors:** Tamar Paz-Elizur, Yael Leitner-Dagan, Kerstin B Meyer, Barak Markus, Federico M Giorgi, Martin O’Reilly, Hyunjin Kim, Yentl Evgy, Ronen Fluss, Laurence S Freedman, Robert C Rintoul, Bruce Ponder, Zvi Livneh

**Affiliations:** 1 Department of Biomolecular Sciences, Weizmann Institute of Science, Rehovot, Israel; 2 Cancer Research UK Cambridge Institute, University of Cambridge, Cambridge, UK; 3 Department of Oncology, University of Cambridge, Cambridge, UK; 4 Bioinformatics Unit, Grand Israel National Center for Personalized Medicine, Weizmann Institute of Science, Rehovot, Israel; 5 Biostatistics Unit, Gertner Institute for Epidemiology and Public Health Policy Sheba Medical Center, Tel Hashomer, Israel; 6 Department of Pharmacy and Biotechnology, University of Bologna, Bologna, Italy; 7 Department of Thoracic Oncology, Royal Papworth Hospital, Cambridge, UK

## Abstract

**Background:**

Improving lung cancer risk assessment is required because current early-detection screening criteria miss most cases. We therefore examined the utility for lung cancer risk assessment of a DNA Repair score obtained from OGG1, MPG, and APE1 blood tests. In addition, we examined the relationship between the level of DNA repair and global gene expression.

**Methods:**

We conducted a blinded case-control study with 150 non–small cell lung cancer case patients and 143 control individuals. DNA Repair activity was measured in peripheral blood mononuclear cells, and the transcriptome of nasal and bronchial cells was determined by RNA sequencing. A combined DNA Repair score was formed using logistic regression, and its correlation with disease was assessed using cross-validation; correlation of expression to DNA Repair was analyzed using Gene Ontology enrichment.

**Results:**

DNA Repair score was lower in case patients than in control individuals, regardless of the case’s disease stage. Individuals at the lowest tertile of DNA Repair score had an increased risk of lung cancer compared to individuals at the highest tertile, with an odds ratio (OR) of 7.2 (95% confidence interval [CI] = 3.0 to 17.5; *P* < .001), and independent of smoking. Receiver operating characteristic analysis yielded an area under the curve  of 0.89 (95% CI = 0.82 to 0.93). Remarkably, low DNA Repair score correlated with a broad upregulation of gene expression of immune pathways in patients but not in control individuals.

**Conclusions:**

The DNA Repair score, previously shown to be a lung cancer risk factor in the Israeli population, was validated in this independent study as a mechanism-based cancer risk biomarker and can substantially improve current lung cancer risk prediction, assisting prevention and early detection by computed tomography scanning.

Lung cancer is the leading cause of cancer deaths, being responsible for about 156 000 deaths per year in the United States (1,2), and 388 000 deaths per year in Europe (3). Detection of lung cancer at an early stage dramatically increases the 5-year survival rate from less than 5% at stage IV to 54%–73% at stage I, highlighting the effectiveness of early detection in reducing lung cancer mortality (4). Identification of people at high risk for lung cancer is important for empowering effective early detection, and currently the main lung cancer risk factors are age and smoking history (5–9). Indeed, recommendations for computed tomography (CT) screening for early lung cancer detection in the United States are based on age and heavy smoking, which were used in the National Lung Screening Trial (NLST) (10). This trial showed that CT screening followed by treatment led to a 20% reduction in lung cancer mortality (10), confirmed by the NELSON trial, which recently showed a 26% reduction in lung cancer mortality in men after CT screening and treatment (11), and consistent with the earlier single-arm I-ELCAP study (12). If the NLST selection criteria were to be applied to all eligible individuals in the United States, estimations show that only 27% of lung cancer case patients would be detected (13). Although this is a substantial proportion, it does suggest that additional risk factors await discovery and employment.

Because DNA repair is crucial for removing DNA damage and avoiding mutations (14–16), suboptimal DNA repair is likely to cause cancer risk. This is clearly illustrated in the extreme cases of germline mutations in DNA Repair genes, which cause repair deficiency and high predisposition to cancer (17–19). Similarly, suboptimal DNA Repair activity was reported to be associated with cancer risk for a variety of DNA Repair mechanisms and cancer types (20–25).

Here we examined, in a blinded, case-control study, whether a low DNA Repair score, calculated from the DNA Repair enzymatic activities of OGG1 (8-oxoguanine DNA glycosylase), MPG (methylpurine DNA glycosylase), and APE1 (apurinic/apyrimidinic endonuclease 1) (also known as OMA score), all of which act primarily on oxidative DNA damage, is a risk biomarker for lung cancer in the United Kingdom as it was in the Israeli population (23,25–27). In addition, we examined whether variation in the DNA Repair score measured in peripheral blood mononuclear cells (PBMC) correlates with systematic effects on gene expression in airway cells of lung cancer patients and control individuals.

## Methods

### Study Participants

Patients at least age 18 years referred to Royal Papworth Hospital, Cambridge, Thoracic Oncology service for investigation of suspected lung cancer were recruited between October 2013 and November 2015. Those with a history of previous lower or upper airway cancer were excluded, as were patients with symptoms who were initially recruited to the study but later were found to have no evidence of cancer. Control individuals were healthy volunteers recruited from the Cambridge BioResource, a panel of approximately 17 600 volunteers, both with and without health conditions, who are willing to be approached to participate in research studies (https://www.cambridgebioresource.group.cam.ac.uk). They were selected in an attempt to form a sex and age composition comparable to the cases group. Disease stage was reported according to the seventh edition of the tumor, lymph node, metastasis (TNM) classification of malignant tumors (28). Research ethics approvals for sample collection from participants in this study were given by East of England Cambridge Central REC 13/EE/0012 and the National Research Ethics Service Committee South East Coast – Surrey 13/LO/0889. Table 1 summarizes the characteristics of case patients and control individuals.

**Table 1.** Characteristics of case patients and control individuals

Characteristics	Control individuals (N = 143)	Case patients (N = 150)	*P* *
Sex, No. (%)			.291
Male	79 (55.2)	93 (62.0)	
Female	64 (44.8)	57 (38.0)	
Age, mean (SD)	59.7 (10.0)	68.6±9.6	<.001
Smoking status, No. (%)			<.001
Current	48 (33.6)	62 (41.3)	
Ex-smoker	50 (35.0)	87 (58.0)	
Never	45 (31.5)	1 (0.7)	
Pack-years, mean (SD)	16.8±20.3	43.2±27.6	<.001
Histology, No. (%)			
Adenocarcinoma	—	83 (55.3)	
Squamous	—	56 (37.3)	
NSCLC-NOS	—	11 (7.3)	
COPD, No. (%)			<.001
None	112 (83.6)	56 (41.8)	
Mild	9 (6.7)	26 (19.4)	
Moderate	10 (7.5)	31 (23.1)	
Severe	3 (2.2)	16 (11.9)	
Severity unknown	0 (0.0)	5 (3.7)	

* *P* value comparing the distribution of case patients with control individuals: For categorial values the chi-square test was used; for continuous variables the unpaired t test was used. COPD = chronic obstructive pulmonary disease; NSCLC-NOS = non–small cell lung cancer–not otherwise specified; — = not applicable; Missing values: pack-years: three control individuals; COPD: nine control individuals and 16 case patients.

### Specimens, Protein Extracts, DNA Repair Assays, and RNA Sequencing

**Figure 1.**
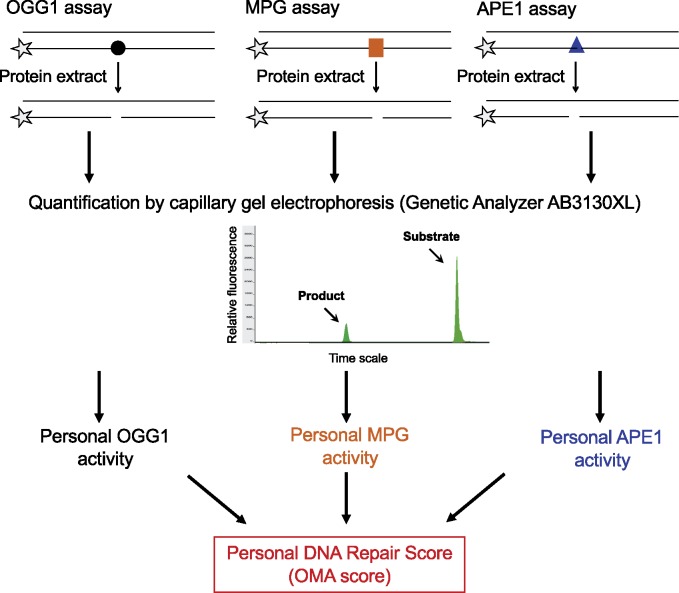
Outline of the panel of DNA Repair assays used to determine the personal DNA Repair score. Each assay measures the activity of a specific DNA Repair enzyme, in a protein extract prepared from PBMC, to remove its target DNA damage from a synthetic short oligonucleotide, shown schematically in the upper panel. Each enzyme assay was run separately under optimized conditions. The target damaged base in each substrate oligonucleotide was 8-oxoguanine in the OGG1 assay (illustrated by a **black circle**), hypoxanthine in the MPG assay (**orange square**), and a furanyl abasic site in the APE1 assay (**blue triangle**). The asterisks represent a 3'-Yakima Yellow fluorescence tag. Assays were run on a robotic platform and analyzed in an AB3130XL genetic analyzer. The personal OGG1, MPG, and APE1 enzyme activities were then used to calculate the personal DNA Repair score for each participant, as described in Methods. Further description of the DNA Repair assays is provided in the Supplementary Materials (available online). APE1 = apurinic/apyrimidinic endonuclease 1; MPG = methylpurine DNA glycosylase; OGG1 = 8-oxoguanine DNA glycosylase; OMA = OGG1, MPG, and APE1; PBMC = peripheral blood mononuclear cells.

Each participant donated 17 mL blood (two 8.5 mL ACD vacutainers) from which, within 2–4 hours, PBMC were isolated by Ficoll fractionation and frozen as previously described (23,29). Protein extracts, DNA substrates, and DNA Repair assays were performed as previously described (24,25,30,31) and are briefly described in the Supplementary Methods (available online). The outline of the DNA Repair assays is described in Figure 1. Bronchial and nasal sample collection, along with RNA extraction, sequencing, and analysis, are described in the Supplementary Methods (available online).

### Statistical Analysis

Personal characteristics of case patients and control individuals were compared using unpaired *t* tests for continuous variables and χ^2^ tests for categorical variables. The smoking status distribution differed between case patients and control individuals, with approximately 30% of the control individuals being never smokers compared to only one case patient. In addition, whereas case patients and control subjects were quite well-matched by sex, the control individuals were approximately 9 years younger than the case patients on average. Therefore, all comparisons of case patients and control individuals were adjusted for smoking and age, as well as sex. The adjustment was performed by using regression models in which these factors were included as covariates. Because of the large imbalance in the smoking variable, these regression analyses essentially compare the 98 ever-smoked control individuals with the 149 ever-smoked case patients, and little information is gained from the never-smokers. Indeed, in additional analyses where we excluded the never-smokers, the regression results were virtually unchanged from those reported in Results. Chronic obstructive pulmonary disease (COPD) was reported more commonly among the lung cancer case patients than among the control individuals; no adjustment for COPD was made in our analyses because it is likely a mediating factor rather than a confounder in the association of DNA Repair enzymes with lung cancer, and adjusting for mediating factors is not recommended (32).

The association of enzymatic DNA repair activity levels with lung cancer risk was evaluated using an unconditional logistic regression model in which the presence or absence of lung cancer was the binary dependent variable, and the covariates were the DNA Repair enzyme levels (OGG1, MPG, and APE1) together with age (continuous), sex, and smoking status (smoker, past smoker, never smoker) as adjusting variables. From this model, the DNA Repair scores were derived using the estimated regression coefficients for the OGG1, MPG, and APE1 variables. The resulting formula of the DNA Repair score for each study participant was DNA Repair score = 0.00621 × APE1 – 0.047 × OGG1 – 0.0223 × MPG. Examination of the scores for case patients and control individuals showed them to be approximately normally distributed within each group. This score was then entered as a covariate in a new logistic regression model, either as a continuous variable or in tertile categories (according to the score distribution in the control individuals), with the same adjusting variables, to obtain odds ratio estimates and receiver-operating characteristic (ROC) curves to describe the strength of the DNA Repair score–lung cancer association. The DNA Repair score formula optimized for this cohort of participants was different from the previous study (25), most likely because of the revised APE1 assay in this study and the fact that different cohorts of modest size were used in the two studies. To overcome the bias arising from use of the same data to develop the DNA Repair score and evaluate its strength of association, the odds ratios and ROC curves were reestimated using “leave-one-out” cross-validation (33). Confidence limits (bias-correction and acceleration method) for the area under the curve (AUC) of the ROC and the comparison test of two AUCs were computed using the nonparametric bootstrap. The continuous scores were also compared with case-control status, age group (≤55, 56–69, ≥70 years), sex, smoking status, and histology and disease stage (among case patients) using analysis of covariance, in each case adjusting for the other factors (Table 2). A *P* value equal to or less than .05 was considered statistically significant and all tests were two-sided.

**Table 2.** Distribution of selected characteristics and DNA Repair score* between non–small cell lung cancer case patients and control individuals

	Control individuals (n = 140)			Case patients (n = 149)	
Characteristics	No.	DNA Repair score mean (95% CI)	*P*	No.	DNA Repair score mean (95% CI)
All†	140	4.00 (3.84 to 4.16)		149	2.67 (2.50 to 2.84)
			*P*_Case vs control_‡ < .001		
Histology					
SQCC	–	–		56	2.69 (2.45 to 2.93)
Adenocarcinoma	–	–		82	2.62 (2.38 to 2.86)
Unknown	–	–		11	2.92 (1.87 to 3.98)
			*P*_SQCC vs adenocarcinoma_§ = .65		
Age, y					
≤55	43	4.28 (3.96 to 4.59)		16	2.36 (1.82 to 2.89)
56–69	75	4.00 (3.81 to 4.20)		64	2.86 (2.55 to 3.16)
≥70	22	3.45 (2.95 to 3.94)		69	2.56 (2.35 to 2.78)
			*P*_Age trend_‖ = .075		
Sex					
Male	77	3.82 (3.59 to 4.05)		93	2.71 (2.49 to 2.93)
Female	63	4.21 (3.99 to 4.44)		56	2.60 (2.32 to 2.88)
			*P*_M vs F_‖ = .47		
Smoking status					
Never smoked	45	4.07 (3.79 to 4.36)		1	4.85
Past smoker	49	3.98 (3.72 to 4.24)		87	2.60 (2.40 to 2.81)
Current smoker	46	3.95 (3.62 to 4.27)		61	2.72 (2.43 to 3.02)
			*P*_Ever-smoked vs never smoked_‖ = .32;		
			*P*_Current smoker vs others_‖ = .65		
COPD					
No	109	4.02 (3.85 to 4.19)		56	2.86 (2.55 to 3.18)
Yes	22	3.82 (3.30 to 4.34)		77	2.57 (2.35 to 2.79)
			*P*_Yes vs no_¶ = .13		

* The DNA Repair score was defined as: 0.00621 × APE1 − 0.047 × OGG1 − 0.0223 × MPG, where APE1, OGG1, and MPG each represent the measured enzyme activity of an individual. The weights for each component used in the DNA Repair score were calculated from the logistic regression and chosen to optimize strength of association of the score with lung cancer for the observed data set. APE1 = apurinic/apyrimidinic endonuclease 1; CI = confidence interval; COPD = chronic obstructive pulmonary disease; MPG = methylpurine DNA glycosylase; OGG1 = 8-oxoguanine DNA glycosylase; SQCC = squamous cell carcinoma.

† Values were missing for three control individuals (two current and one past smoker) and one case patient (current smoker).

‡ Two-sided *P* value comparing the distribution of case patients with control individuals: analysis of covariance was used adjusting the comparison for continuous age, sex, and smoking status.

§ Two-sided *P* value for comparing SQCC with adenocarcinoma: analysis of covariance was used adjusting for case-control-status, continuous age, sex, and smoking status.

‖ Two-sided *P* values obtained from analysis of covariance for factor of interest, adjusted for case-control status and other factors from among age, sex, and smoking status.

¶ Two-sided *P* value comparing COPD vs no COPD; using analysis of covariance adjusting for continuous age and sex.

## Results

### DNA Repair Score in Lung Cancer Patients Compared to Control Individuals in the United Kingdom

**Figure 2.**
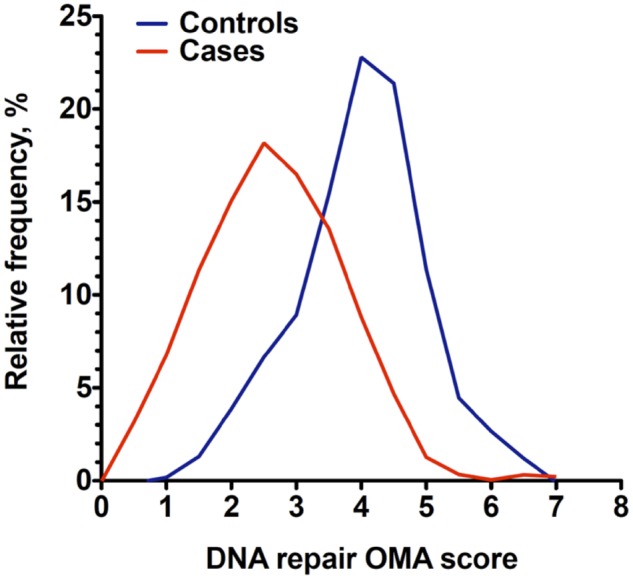
DNA Repair score distribution among lung cancer case patients and control individuals. The frequency distribution of DNA Repair scores is shown for 149 lung cancer patients (**red curve**) and 140 control individuals (**blue curve**). The graphs were plotted using GraphPad Prism version 5.00 (GraphPad Software, San Diego, CA), with bin width automatically chosen by the software, with second- order smoothing, with two neighbors on each size. Bin width was 0.5 units. APE1 = apurinic/apyrimidinic endonuclease 1; MPG = methylpurine DNA glycosylase; OGG1 = 8-oxoguanine DNA glycosylase; OMA = OGG1, MPG, and APE1.

To examine whether a low DNA Repair score is associated with lung cancer risk in the United Kingdom, we conducted a case-control study with 150 non–small cell lung cancer case patients and 143 control individuals for whom we tested the enzymatic activities for OGG1, MPG, and APE1 in PBMC. Participant accrual, blood collection, and PBMC isolation were performed in the United Kingdom, after which the PBMC samples were coded and sent unidentified to Israel to determine their DNA Repair activities. After completion of DNA Repair testing, the study was unblinded, and the clinical and DNA Repair data were used for the statistical analysis. From the logistic regression relating levels of OGG1, MPG, and APE1 to lung cancer risk (adjusting for age, sex, and smoking status), the DNA Repair score (defined in the footnote to Table 2) was derived. Results of the analysis of this DNA Repair score are presented in Table 2 and Figure 2. The mean score in patients was 2.67 (95% confidence interval [CI] = 2.50 to 2.84) and was lower than in the control individuals: 4.00 (95% CI = 3.84 to 4.16; *P* < .001; Table 2). Consistently, the distribution of DNA Repair score values among patients was shifted to lower values compared to control individuals (Figure 2). There were no statistically significant differences between the mean scores of men and women; individuals with or without COPD; never, past, and current smokers; and patients with adenocarcinoma or squamous cell carcinoma (Table 2). There appeared to be a decrease in DNA Repair score with age among control individuals, but it did not reach statistical significance (Table 2).

### Association Between Low DNA Repair Score and Lung Cancer

When the DNA Repair score was assessed as a continuous variable in relation to lung cancer risk, a 1-SD decrease in the DNA Repair score was associated with an increased adjusted odds ratio of 2.7 (95% CI = 1.9 to 3.7; *P* < .001), and 2.5 (95% CI = 1.8 to 3.4; *P* < .001) after cross-validation (Table 3). When the score was categorized into tertiles, using the tertile with the highest score as reference, the adjusted odds ratio for lung cancer in the lowest tertile was 11.5 (95% CI = 4.3 to 31.2; *P* < .001), and 7.2 (95% CI = 3.0 to 17.5; *P* < .001) after cross-validation (Table 3). These results suggest that a low DNA Repair score is strongly associated with lung cancer risk.

**Table 3.** Unconditional logistic regression analysis of the DNA Repair score*

Variable	Score value	No. of control individuals (%)	No. of case patients (%)	Adjusted OR† (95% CI)	*P*	Cross-validation Adjusted OR‡ (95% CI)	*P*
Score per 1 SD decrease§	0.98 U	140 (100.0)	149 (100.0)	2.7 (1.9 to 3.7)	<.001	2.5 (1.8 to 3.4)	<.001
Score by tertiles‖							
Highest tertile	≥4.52	47 (33.6)	6 (4.0)	1.0 (Referent)	—	1.0 (Referent)	—
Middle tertile	3.68–4.51	46 (32.9)	22 (14.8)	3.0 (1.0 to 8.7)	.048	1.6 (0.6 to 4.2)	—
Lowest tertile	≤3.68	47 (33.6)	121 (81.2)	11.5 (4.3 to 31.2)	<.001	7.2 (3.0 to 17.5)	—

* DNA Repair score definition: 0.00621 × APE1 – 0.047 × OGG1 – 0.0223 × MPG. APE1 = apurinic/apyrimidinic endonuclease 1; CI = confidence interval; MPG = methylpurine DNA glycosylase; OGG1 = 8-oxoguanine DNA glycosylase; OR = odds ratio.

† Unconditional logistic regression adjusted for age, sex, and smoking status (smoker, past smoker, never smoker).

‡ Cross-validated odds ratios calculated according to the SDs and tertiles of the cross-validated DNA Repair scores.

§ Score was fitted in the unconditional logistic regression model as a continuous variable. 0.98 U is the 1 SD in the control group for the DNA Repair scores. For each model, the estimated odds ratios for smoking were slightly different and the range was as follows: current vs never = 57.1–59.4; current v ex = 1.27–1.29.

‖ Tertiles of the control individuals’ values. The upper tertile was chosen as the referent.

### Patients' DNA Repair Score by Disease Stage Compared to Healthy Individuals

**Figure 3.**
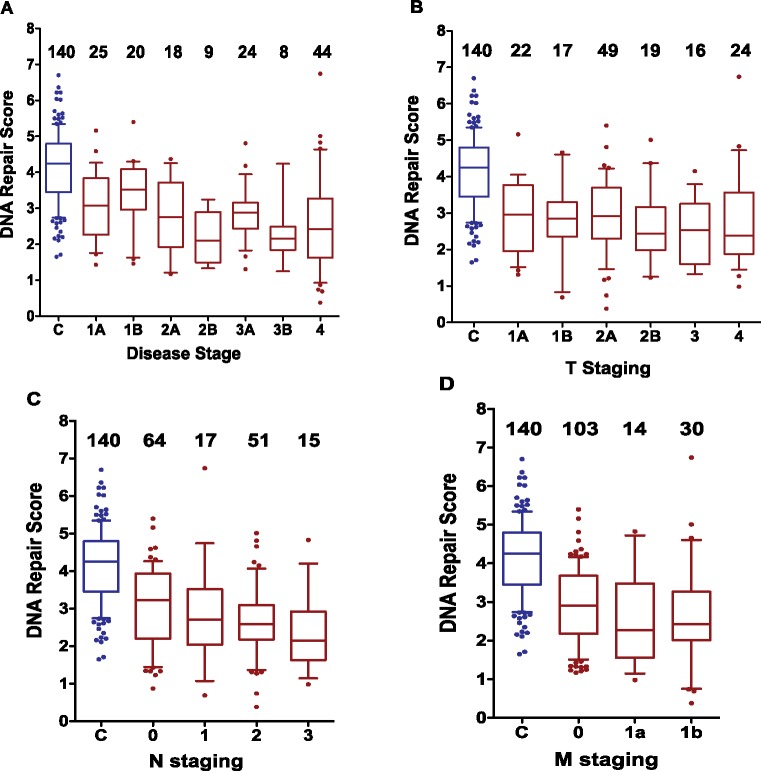
Analysis of the DNA Repair scores by disease staging. The distributions of the DNA Repair scores are presented for the control individuals **(C blue)** and for the lung cancer patients by stage status (in **red**) using boxplots (whiskers 10th–90th percentile) showing the values lower than the first 10th percentile and greater than the 90th percentile as dots, with the line in the middle plotted at the median. Distributions are presented by disease state **(A)**, T staging **(B**), N staging **(C)**, and M staging **(D)**. The number of participants in each subgroup is indicated above the plots. Importantly, the differences between the control groups and each of the earliest stagings in each category is statistically significant. C vs disease stage IA, *P *=* *.0009; C vs T1A, *P *=* *.011; C vs N0, *P *<* *.0001; C vs M0, *P *<* *.0001. All *P* values were adjusted for sex, age, and smoking status. See related numerical values in Supplementary Table 1 (available online), where the means and SDs are presented. The graphs were plotted using GraphPad Prism version 5.00 (GraphPad Software, San Diego, CA).

The DNA Repair score for case patients was examined within each disease stage and each TNM category. The mean score in patients with stage IA lung cancer was 2.95 (95% CI = 2.56 to 3.35), statistically significantly lower than the mean of control individuals (4.00, 95% CI = 3.84 to 4.16; *P* = .0009), adjusted for age, sex, and smoking status (Figure 3 and Supplementary Table 1, available online). Within TNM categories, the mean DNA Repair scores in lung cancer patients with T1A, N0, and M0 were 2.80 (95% CI = 2.35 to 3.25), 2.90 (95% CI = 2.64 to 3.17), and 2.76 (95% CI = 2.58 to 2.95), respectively, each statistically significantly lower than in control subjects (DNA Repair score = 4.00), with *P* values of .011 for the first, and less than .0001 for the latter two comparisons. These findings are consistent with low DNA Repair score being a risk factor for lung cancer, possibly acting from the early stages of lung carcinogenesis. The tests performed show a further small yet statistically significant decline in the DNA Repair score for more advanced disease stage, N category and M category, and there is also a statistically nonsignificant decline over T categories (Supplementary Table 1, available online).

### Sensitivity, Specificity, and ROC of DNA Repair Score Testing

**Figure 4.**
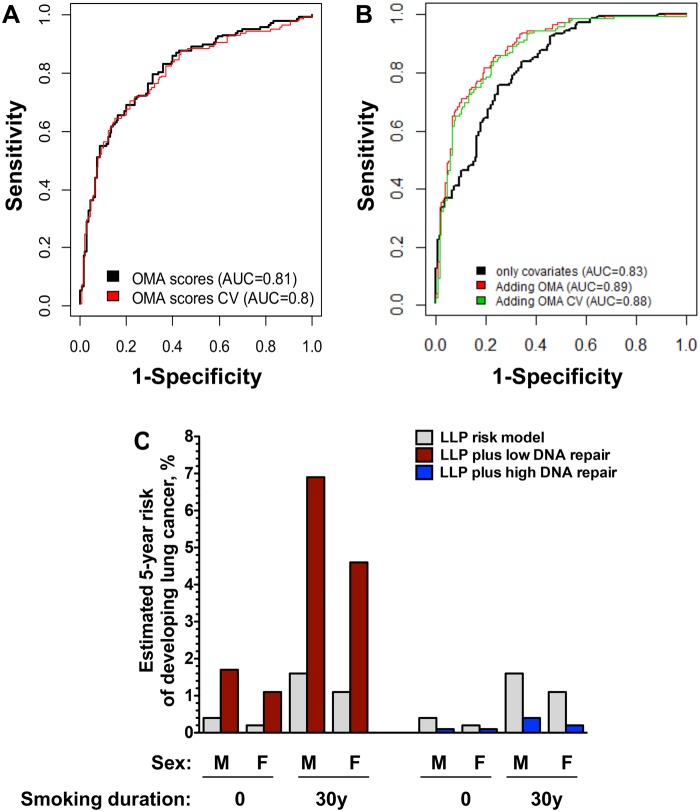
Receiver operating characteristic (ROC) curve of the sensitivity and specificity of the DNA repair score in lung cancer risk and examples of its added value. **A)** ROC curve for the DNA repair scores obtained in the current study (**black curve**) and after cross-validation (**red curve**). **B)** ROC curve for the covariates age, sex, and smoking status (**black curve**) and after adding the DNA repair score to these basic covariates before (**red curve**) and after (**green curve**) cross-validation. **C)** Estimated added value of the DNA repair score to lung cancer risk estimates based on the Liverpool Lung Project (LLP) Risk Model. Estimates are presented for a man and a woman, age 65 years, who are either never smokers or smoked for 30 years. **Gray columns** represent the 5-year risk according to the Lung Project Risk model. The effect of having a low DNA repair score of 5th percentile or less (**red columns**), or a high DNA repair score of 75th percentile or greater (**blue columns**) are presented. Data were taken from Supplementary Table 4 (available online). APE1 = apurinic/apyrimidinic endonuclease 1; AUC = area under the curve; CV = cross-validated; F = female; M = male; MPG = methylpurine DNA glycosylase; OGG1 = 8-oxoguanine DNA glycosylase; OMA = OGG1, MPG, and APE1.

Plotting the sensitivity and specificity of the DNA Repair score combined with age and smoking status in ROC curves yielded an AUC of 0.89 (95% CI = 0.82 to 0.93) and 0.88 after cross-validation (Figure 4B). When DNA Repair score was assessed separately from age and smoking, the AUC was 0.81 for the DNA Repair score (0.80 after cross-validation; Figure 4A). The AUC for age and smoking status without the DNA repair score was 0.83 (95% CI = 0.75 to 0.89) (Figure 4B). The added value of the DNA Repair score in estimating lung cancer risk on top of the risk predicted based on age and smoking was statistically significant (*P* = .0002) and is illustrated by the following sensitivity values: at a specificity of 95%, the sensitivity of smoking and age as risk factors is 34%, with the DNA Repair score exhibiting a similar independent sensitivity of 36%. When the two are combined, the sensitivity is increased to 54%. Similarly, at a specificity of 90%, the combined sensitivity is 64%, higher than smoking and age (43%) or DNA Repair score (50%) risk factors alone.

### Correlation Between DNA Repair Score and Expression of Biological Pathways in Lung Cancer Patients

Having the DNA Repair scores of a group of people, we sought to examine whether interpersonal variations in DNA repair correlate with systemic effects in the human body. To that end, we examined the relationship between the DNA Repair score and whole transcriptome RNAseq analysis in airway epithelial cells. Analysis included 213 participants (92 healthy control individuals and 121 lung cancer patients) selected after performing quality control procedures and data cleaning on a larger RNAseq dataset (see Supplementary Methods, available online). The correlation between RNA expression levels and DNA Repair score was tested separately for case patients and control subjects, by regressing the RNA expression levels of each gene in the dataset on the DNA Repair score values using the DESeq2 tool (34). Sex, age, smoking status, and experimental batch were incorporated as adjusting variables. Few genes were statistically significantly correlated with scores at a false discovery rate threshold of 0.01 (Supplementary Methods, available online).

**Figure 5.**
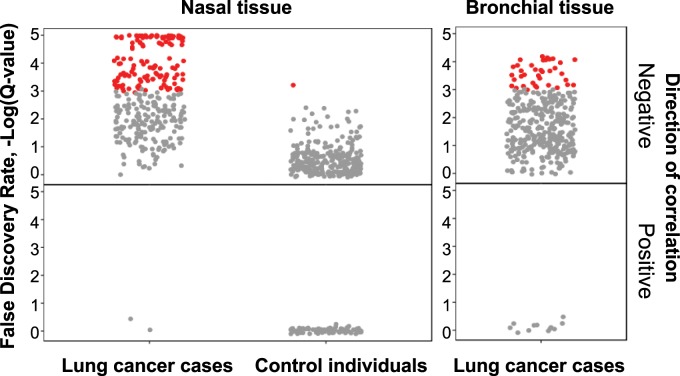
Enrichment of immune system related pathways with low DNA Repair score using gene set enrichment analysis (GSEA). Genes ranked by the RNAseq2 analysis according to their correlation to the DNA Repair score were analyzed by GSEA to identify pathways (using gene ontology terms) that statistically significantly correlate with the DNA Repair score. The analysis was performed separately for case patients and control individuals. This leads to a list of pathways ranked according to their normalized enrichment score, the top 30 of which are shown in Supplementary Table 2 (available online). The figure presents for each tissue and health state all of the 366 immune system–related pathways as found by the GSEA analysis (according to keywords presented in Supplementary Table 2, available online). Each dot represents a pathway, with its *y-*axis value showing the false discovery rate, -Log(Q-value), for the enrichment score. The pathways are colored according to their false discovery rate values: **gray dots** Q-Value > 0.001, **red dots** Q-Value ≤ 0.001. Negatively and positively enriched pathways are shown for nasal and bronchial tissues, in case patients and control individuals, as indicated.

Hypothesizing that there is a signal in the data that is distributed over many genes, with small effect size in each gene, we examined whether there are pathways that are statistically significantly enriched with varying score values, using Gene Set Enrichment Analysis (GSEA) (35). For each group (case patients and control subjects), we ranked approximately 15 000 genes based on the statistics of DESeq2 and used this ranking as input to GSEA with default parameters. We tested for overrepresentation of genes belonging to specific pathways as defined by gene ontology terms (Supplementary Tables 2 and 3, available online). Pathways identified by GSEA were manually curated and divided into three groups: immune system-related pathways, cell cycle pathways, and other pathways (see keywords in legend to Supplementary Table 3, available online). The analysis revealed 185 immune response pathways that were statistically significantly enriched, spanned by 3154 unique genes negatively correlated with the DNA Repair score. Strikingly, this correlation was specific for the lung cancer patients and was not observed in the control individuals’ samples (Figure 5, Supplementary Table 3, available online). The dramatic enrichment in the immune response pathways was robust against subsampling, indicating that it is not a sampling bias (Supplementary Methods and Supplementary Figure 3, available online).

Another set of pathways that exhibited negative correlation with the DNA Repair score represents cell cycle pathways (Supplementary Table 3, available online). This correlation was found both in lung cancer patients and control individuals, with a larger number of pathways enriched in the control participants. RNAseq analysis of 37 bronchial samples, which were obtained only from case patients, also showed an enrichment of immune system–related pathways (Figure 5). In conclusion, the expression data suggest that low DNA Repair score correlates with broad upregulation of immune response pathways in lung cancer patients, but not in control individuals.

## Discussion

### 

The results presented here indicate that a personal low baseline DNA Repair score, composed of the enzymatic activities of OGG1, MPG, and APE1, is a strong risk factor for lung cancer. These results validate a previous study performed in an Israeli population (25) and exhibit comparable estimated relative risk values (Supplementary Figure 4, available online), suggesting that low DNA Repair score is a general lung cancer risk factor in different countries and populations.

Although the studies were retrospective, several lines of evidence suggest that a low DNA Repair score may play a causative role in lung cancer: DNA Repair score is lower in patients at any disease staging (overall disease stage and each TNM staging separately) than in control individuals, including stage I disease, consistent with low personal baseline DNA repair contributing to the carcinogenesis process; We have previously compared DNA Repair activity in blood samples before and after surgery of non–small cell lung cancer patients and found no correlation between DNA Repair activity and the presence of the tumor (27); In a study on the role of low DNA Repair in the risk of head and neck cancer, we found that DNA Repair activity at diagnosis, and 3–4 years after treatment and cure, was similar within the 95% confidence interval (26). This suggests that baseline DNA repair is a personal feature characteristic of an individual and is not affected by the disease; and the general well-established role of DNA repair is eliminating DNA damage and avoiding mutations and cancer (36).

Nevertheless, to definitively prove the causative role of the DNA Repair biomarker in lung cancer, a prospective study is needed.

### 

Screening for early detection of lung cancer based on the NLST criteria (age, heavy smoking) is expected to apply to about 7.5% of all smokers and ex-smokers in the United States and detect about 27% of lung cancers (13). This leaves 92.5% of smokers and former smokers in the United States and 73% of lung cancer case patients with no proper risk assessment for early detection of lung cancer. Our results suggest that adding the DNA Repair score to the age and smoking considerations is likely to improve risk assessment of individuals. For example, using the Liverpool Lung Project model (see Data Supplements for the calculation, available online), a male aged 65 years, having smoked for 30 years with no other risk factors, has a projected 1.6% 5-year risk of developing lung cancer (37). However, assuming the person also has a low DNA Repair score (≤5th percentile), his risk increases approximately fourfold to 6.9% (Figure 4C;Supplementary Table 4, available online). Conversely, if the person had smoked for 50 years, then his projected risk would be 4.5%, but knowing that the person had a relatively high DNA Repair score (≥75th percentile), his risk would drop to 1.1% (Supplementary Table 4, available online). These results illustrate how using the DNA Repair score could greatly improve lung cancer risk assessment, aiding selection of patients for lung cancer early detection and paving the way to broadening primary and secondary prevention of lung cancer beyond the NLST criteria.

### 

Aside from the association of low DNA Repair score with lung cancer risk, we observed a striking correlation between low DNA Repair score and a broad activation of immune-related pathways in lung cancer patients, measured by RNAseq gene expression (Supplementary Tables 2 and 3, available online). The correlation withstood two types of robustness tests, indicating that the effect did not occur by chance. Why was such an effect not observed in control individuals? It is possible that activation of the immune system requires the presence of cancer cells or cells presenting neoantigens, and in addition, the level of DNA repair was lower in case patients than in control individuals. A low DNA repair might cause a higher mutational burden and an increase in neoantigens, contributing to the activation of the immune system (38,39), similar to the situation in tumors with a mismatch repair deficiency (40–42). In addition, the tested DNA Repair enzymes affect the immune system via other mechanisms (43–47). Because only about 1% of lung cancer case patients exhibit a mismatch repair deficiency (48,49), a low DNA Repair score might serve as a biomarker for response to immunotherapy of these patients, but of course this needs to be directly tested.

The data obtained thus far indicate that DNA Repair score testing may provide a highly useful risk biomarker that when combined with smoking history and age can provide effective risk estimates for lung cancer, beyond the classical smoking history and age risk factors (Figure 4C). Such risk assessment may have applications in referring low-dose CT screening to high-risk individuals who are ineligible under current NLST criteria and aiding management of indeterminate pulmonary nodules, assisting prevention, early detection, and therapy of lung cancer.

## Funding

This work was funded by grants from NIH/NCI/EDRN (#1 U01 CA111219), the Flight Attendant Medical Research Institute, Florida, the Mike Rosenbloom Foundation and Weizmann Institute of Science to ZL and TPE; and by grants from Cancer Research UK to BP and to the Cancer Research UK Cambridge Centre; and by a UK National Institute for Health Research Senior Fellowship to BP; and by the Cambridge Biomedical Research Centre and the Cancer Research UK Cambridge Centre to RCR. Volunteer participant recruitment through the Cambridge Bioresource was funded by the Cambridge Biomedical Research Centre.

## Notes

Affiliations of authors: Department of Biomolecular Sciences, Weizmann Institute of Science, Rehovot, Israel (TPE, YLD, YE, ZL); Cancer Research UK Cambridge Institute, University of Cambridge, Cambridge, UK (KBM, FMG, MOR, HK, BP); Department of Oncology, University of Cambridge, Cambridge, UK (KBM, FMG, MOR, HK, RCR, BP); Bioinformatics Unit, Grand Israel National Center for Personalized Medicine, Weizmann Institute of Science, Rehovot, Israel (BM); Biostatistics Unit, Gertner Institute for Epidemiology and Public Health Policy Sheba Medical Center, Tel Hashomer, Israel (RF, LSF); Department of Pharmacy and Biotechnology, University of Bologna, Bologna, Italy (FMG); Department of Thoracic Oncology, Royal Papworth Hospital, Cambridge, UK (RCR).

We gratefully acknowledge the participation of all NIHR Cambridge BioResource volunteers, and thank the NIHR Cambridge BioResource Centre and staff for their contribution. We thank the National Institute for Health Research and NHS Blood and Transplant.

We thank Dr Brendan Dougherty, Dr David Meek, Dr Nick Carroll, Dr Nicky Simler, Amanda Stone, Anne Joy, Tania Pettit, Ann Thomson, Victoria Senior, Theresa Green, Amy Gladwell for subject recruitment, and data and sample collection; Tim Young, Julia Knight, and Natalie Allen for excellent technical assistance in sample processing; the study participants, for enabling us to perform this study; and Prof Eytan Domany (Department of Physics of Complex Systems, Weizmann Institute of Science) for his advice on the bioinformatics analysis.

Conflict of interest statement. The corresponding authors (ZL and TPE) have an issued patent on the OGG1 risk factor for lung cancer and patent applications issued and pending for the OGG1, MPG, and APE1 panel of DNA Repair biomarkers. The other authors declare no conflict of interest.

The funders had no role in the design of the study; the collection, analysis, and interpretation of the data; the writing of the manuscript; and the decision to submit the manuscript for publication.

## Supplementary Material

pkz067_Supplementary_Data
