# Supplementary material for: DNA Repair Biomarker for Lung Cancer Risk and its Correlation With Airway Cells Gene Expression
Source: JNCI Cancer Spectr. 2019 Sep 12;4(1):pkz067. doi: 10.1093/jncics/pkz067 (PMC7012022; doi:10.1093/jncics/pkz067)
Supplement: pkz067_Supplementary_Data [file pkz067_supplementary_data.pdf]

Online Data – Supplements

DNA-Repair Biomarker for Lung Cancer Risk and its Correlation with Airway Cells Gene Expression

Paz-Elizur et al, 2019

Supplementary Methods

Protein extracts, DNA substrates and DNA repair assays

The DNA repair assays are based on DNA repair nicking assays, in which DNA repair enzymes present in protein extract nick a DNA substrate that contains a site-specific lesion. This converts the full-length substrate into a defined shorter oligonucleotide, and the ratio between the two represents the DNA repair activity (1-4). Protein extracts were prepared by a freeze-thaw protocol, from frozen PBMC that were isolated from blood samples by Ficoll fractionation. Protein concentration was determined using the BCA assay. DNA substrates were synthetic double-stranded DNA oligonucleotides, each carrying a site-specific DNA damage, which is a substrate for the DNA repair enzyme being assayed: 8-oxoguanine for OGG1, hypoxanthine for MPG, and a furanyl abasic site for APE1. Each substrate DNA was 3'-tagged with a Yakima Yellow fluorophore (see Fig. 1 in the manuscript for experimental outline of the panel of DNA repair assays). The enzyme activities of OGG1 and MPG were determined by following the elimination of the damaged base from the DNA, which yielded an abasic site that was further cleaved by the APE1 activity in the extract. Complete cleavage was ensured by treatment with NaOH. APE1 activity was measured by nicking of the substrate at the synthetic abasic site. All assays were conducted using the optimized reaction conditions previously published (1-4), except the APE1 assay, which we revised in this study by reducing the substrate concentration from 40 to 20nM, and shortening reaction time from 15 to 10 minutes. All assays are based on the nicking of the substrate DNA, representing the DNA repair activity. This converts the full-

length substrate into a defined shorter oligonucleotide, and the ratio between the two represents the DNA repair activity. The assays were performed on a robotic platform (Tecan Freedom EVO 200), and the reaction products analyzed by capillary gel electrophoresis, using the ABI3130XL genetic analyzer (Applied Biosystems), and the GeneMapper (Applied Biosystems) and PeakAnalyzer (Robiotec, Rehovot, Israel) software.

Bronchial and nasal sample collection

Bronchial brushings. During diagnostic bronchoscopy procedures three bronchial brushings, designed to gently remove epithelial cells with minimal bleeding, were performed using bronchial brushes (Olympus Medical, Southend, UK). Brushings using disposable cytology brushes (BC-202D-5010 Olympus Japan) were taken from geographically different areas of macroscopically uninvolved main bronchus or lobar bronchi contralateral to the suspected lesion.

Nasal curette samples. Samples of nasal airway epithelium were taken under direct vision from the inferior part of the inferior turbinate of each nostril using nasal curettes (ASI Rhino-Pro; Arlington Scientific Inc.).

RNAseq

Tissue samples from bronchial brushings and nasal curettes were stored in 500μl RNALater overnight at 4°C, and then at -80°C for longer-term storage. RNA was extracted using Qiagen MiRNeasy columns according to manufacturer's protocols. Briefly, bronchial brushes were rinsed in PBS, brushes transferred into 700μl Qiazol and cells lysed by vortexing twice for 30 seconds. For nasal samples the RNALater containing nasal tissue (500μl) was diluted with 2ml of PBS and spun at 10,000 rpm for 10 min. The cell pellet was lysed by resuspension in 700μl Qiazol. For

both types of samples, the Qiazol lysate was applied to a QiaShredder tube (#217004) and spun at 13,000 rpm for 2 mins. The homogenate was kept at room temperature for 5 mins, followed by chloroform extraction using PhaseLock tubes. Nucleic acids in the aqueous phase were precipitated using 1.5 volumes 100% ethanol and DNA was digested using DNAse I. Finally, RNA was isolated from the mixture using RNAeasy mini spin columns. RNA was quantified using a Qbit measurement and quality assessed using an Agilent Bioanalyzer. For samples with a RIN greater than 7, a total of 500ng of RNA was used for Illumina TruSeq Library generation. Sequencing was carried on a HiSeq 2500 Illumina sequencers. Sequencing was carried out in two separate multiplexed experiments. Alignment was carried out on the human genome version GRCh37 using the Tophat alignment tool. On average each library contained above 20 Million reads. Count matrices for cases and controls were processed using DESeq2 (5).

Analysis of RNAseq data

As described in the manuscript, analyzing the relationship between the DNA repair score and gene expression, as determined by RNAseq, uncovered that low DNA repair score correlates with upregulation of immune system pathways in lung cancer patients, but not in control subjects. The following sections describe the methods and results of quality control (QC) procedures, data cleaning and statistical analyses implemented in the main manuscript. The RNAseq dataset included read counts from 669 nasal and bronchial samples derived from 490 subjects, out of which DNA repair score values were available for 213 subjects. The 669 samples' RNAseq dataset (sequencing batches: 494 samples in experiment 1 and 175 in experiment 2) was used in its entirety for the QC analysis.

Quality control analysis of the RNAseq data

Number of detected genes. Gene transcripts were defined as detected if it had counts of more than 10 reads. Genes with <10 reads were filtered out. Samples with less than 13,000 detected genes were filtered out from the analysis. 97.8% of the samples analyzed in experiment 1 and 98.3% in experiment 2 had >13,000 detected genes.

Experimental batches. We used Principal Component Analysis (PCA) to detect the major sources of variation in the data. As expected, tissue type - nasal (NS) versus bronchial (BR), explains most of the variance followed by sequencing batches (experiment 1 versus experiment 2; Supplementary Fig. 1). We did find a few samples that seem to reside in the wrong clusters and removed them from further analysis.

Gender effect. A good quality RNAseq dataset should enable identifying gender differences in gene expression. Stratifying by sequencing batch and tissue type we observed the effect of gender in the PCA of ~100 most variable genes. Several mismatches that were found were removed from further analysis (not shown).

Final number of samples for analysis. Following data cleaning we ended up with 242 samples that had both RNAseq expression data and DNA repair score results: 150 samples from lung cancer patients, including 113 nasal samples (88 from experiment 1 and 25 from experiment 2) and 37 bronchial samples (22 from experiment 1 and 15 from experiment 2), and a total of 92 nasal samples from control subjects (67 from experiment 1 and 25 from experiment 2).

Differential expression analysis

Analysis was performed on the combined dataset obtained from the two experimental batches (Experiments 1 & 2). Similar results were obtained when only the bigger dataset of Experiment 1 was used. The RNAseq data was regressed on DNA repair scores using DESeq2 (5), a regression

tool optimized for RNAseq data. Analysis was performed separately on the different tissues (nasal/bronchial) and disease state (cases / controls), with experimental batch, age, gender, smoking status (never, former and current smokers) and cancer histology (in cases) as adjusting factors. With a False Discovery Rate (FDR) threshold of 0.01, we could find very few genes whose expression correlated with the DNA repair score, as follows: case bronchial samples, 0; case nasal samples, 8; control nasal samples, 1; Nevertheless, it is notable that in the cases group (but not in control subjects) there is an enrichment of genes whose expression increases with decreasing DNA repair OMA score values (left, negative values part of the Volcano plot in Supplementary Fig. 2D). Hypothesizing that the correlation signal might be distributed over many genes, with each gene having a small effect size, we employed gene set enrichment analysis (GSEA; (6)), testing for pathways enriched with genes that are correlated with the DNA repair OMA score.

Gene set enrichment analysis

The list of genes, ranked by their statistics (as reported in DESeq2) was analyzed by GSEA (GSEA 3.0) in order to identify whether there is an over-representation of genes belonging to specific pathways (annotated by Gene Ontology; GO terms; pathway Gene Ontology downloaded from MSigDB (6),c5.all.v6.1). Supplementary Table 2 lists the thirty most significant pathways that were identified. For each pathway, the enrichment algorithm finds the maximum enrichment score, reflecting the degree to which the genes in the set are over-represented at either the top (positive correlation) or bottom (negative correlation) of the list, and calculates the FDR q-value (the false discovery rate), which is the estimated probability that the enrichment score represents a false positive finding. The pathways were manually curated and divided into 3 groups: Immune system-

related pathways, Cell Cycle pathways and Other pathways (see legend to Supplementary Table 3). Supplementary Table 3 summarize the pathways that were found to be significantly enriched in nasal samples by GSEA (q-value<0.001, a very strict value as explained in ref (6)), showing a strong negative association of Immune system-related pathways in the cases group, with essentially no signal in the control groups (see also Fig. 5 in the manuscript). Another set of pathways that exhibited negative correlation with the OMA score represents cell cycle pathways, which unlike the Immune system-related pathways seem to be enriched in both cases and controls (Supplementary Table 3).

To visualize the differences in the correlations between DNA repair score and immune-system pathways, versus DNA repair score and 'Other' pathways, we highlight in differential expression volcano plots two pathways, selected for being relative big and with roughly similar size (~350 genes): the inflammatory response pathway (GO_INFLAMMATORY_RESPONSE, which is an immune system pathway), and the skeletal system development pathway (GO_SKELETAL_SYSTEM_DEVELOPMENT, which belongs to 'Other pathways'). Supplementary Fig. 2 shows Volcano plots, for all the available groups (Cases/Controls)x(Nasal/Bronchial) (in grey dots; Supplementary Fig. 2 A, D, G), highlighting the inflammatory response pathway (in red dots; Supplementary Fig. 2B, E, H) compared to the skeletal system development pathway (in blue dots; Supplementary Fig. 2 C, F, I). The inflammatory response pathway was found to be enriched in the cases both in nasal and in bronchial tissues (left Volcano lobe, Supplementary Fig. 2 E, H), but not in the controls (Supplementary Fig. 2B). The skeletal system development pathway was not enriched in any group/tissue (Supplementary Fig. 2C, F, I).

Simulations to test the robustness of the correlation between a low DNA repair OMA score and activity of immune system pathways

Extreme OMA score trimming analysis. In this section we repeated the analysis for the nasal tissue samples sequenced in experiment 1, except that we excluded samples with OMA scores at the tails of the OMA distribution, removing 3.5% tail from each side of the OMA scores.

The effect of extreme trimming is presented in Supplementary Fig. 3a, showing the upregulation of the immune pathways also with the trimmed OMA score (compare to Fig. 5 in manuscript).

Sub-sampling analysis. To get an estimate for the robustness of the results to a more general sampling noise, we conducted 100 iterations of random sub-sampling of subjects and repeated the regression in each iteration. The RNAseq data of the selected random groups of subjects (at 80% of the sample size) were regressed on OMA scores, followed by gene set enrichment analysis, and the number of significant immune system-related pathways (at a q-value<0.001) was determined. Supplementary Fig. 3b shows that 95% of the simulations have more than 117 significant immune system-related pathways (with median value of 137). This analysis is an indication that the results are not sensitive to sampling noise.

Methods and Tools for RNAseq analysis

All Statistical analysis was conducted in R version 3.2.1 (7). All figures were generated with ggplot2 package (8). Data normalization and regression analysis was done with DESeq2 (5). GSEA and MSigDB (c5.all.v6.1) were used for GO enrichment (6).

Calculation of 5-year risk of lung cancer

The basis of the calculation was the Liverpool Lung Project (LLP) risk model (9). The paper describes a linear logistic regression model for the probability of developing lung cancer within 5 years that depends on several factors: age, sex, smoking duration, prior diagnosis of pneumonia, occupational exposure to asbestos, prior diagnosis of malignant tumor, and family history of lung cancer. To illustrate the effect of the DNA repair score (OMA score) on the risk of lung cancer we did the following: (a) We chose the profile of a male or female aged 65y who had one of the following smoking histories: never smoked, smoked for 10 years, smoked for 30 years or smoked for 50 years, and who had none of the other risk factors in the LP model (i.e. no prior diagnosis of pneumonia, no occupational exposure to asbestos, no prior diagnosis of a malignant tumor and no family history of lung cancer). (b) We assumed that the distribution of OMA DNA repair scores was independent of the risk factors in the LLP model. This is supported by data from the current and previous studies that have shown that the OMA score has small statistically non-significant correlations with age, sex and smoking history. (c) We assumed also that none of the risk factors in the LLP model modify the effect of OMA score on lung cancer risk. This is also supported by data from the current and previous studies that have shown small statistically non-significant interactions between OMA and age, sex and smoking history. (d) Under these assumptions we adapted the LLP model to include the OMA score as an extra factor. The beta-coefficient for the OMA score in this model was log(2.5), where 2.5 was the cross-validated odds ratio estimate for the DNA repair score (see Table 3 of the main paper). For a 65-year old male with the above-mentioned profile, the modified model was:

$$\text{logit}(P) = -5.56 + \text{beta-smok} - \log(2.5) \times (\text{OMA} - 3.553). \qquad (1)$$

In this equation, P is the probability of lung cancer diagnosis within the next 5 years, the value of -5.56 is taken from Table A1 of (9), the value of beta-smok is 0, 0.769, 1.452 or 2.507 respectively for never-smoked, or smoked for 10y, 30y or 50y (taken from Table 2 of (9)), and the value of 3.553 was calculated by us so as to yield an average risk in our control group equal to the average risk in the Liverpool population of males aged 65y in the years 2002-4 (see Table A1 of (9)).

The model for a 65-year old female was that given in Equation (A1) except that -5.56 was replaced by -5.99 (see Table A1 (9)) and 3.553 was replaced by 3.555 (our calculation).

(e) Equation (1) enables the 5-year lung cancer risk to be calculated for a person resident in Liverpool with one of our profiles and a specific value of the OMA score (to be entered into the equation). To calculate the average risk for persons with that same profile but with OMA scores below or above a given percentile (5th, 10th or 75th, as given in Supplementary Table 4), we used numerical integration over the distribution of OMA scores, assuming the DNA repair score had a normal distribution with mean value of 4.00 (see the control group mean in Table 1 in the main manuscript) and standard deviation 0.98 (the control group's SD).

References

1. Leitner-Dagan Y, Sevilya Z, Pinchev M, Kremer R, Elinger D, Rennert HS, Schechtman E, Freedman L, Rennert G, Livneh Z, Paz-Elizur T. Enzymatic MPG DNA repair assays for two different oxidative DNA lesions reveal associations with increased lung cancer risk. Carcinogenesis. 2014;35(12):2763-70. doi: 10.1093/carcin/bgu214. PubMed PMID: 25355292; PMCID: 4303808.

2. Sevilya Z, Leitner-Dagan Y, Pinchev M, Kremer R, Elinger D, Rennert HS, Schechtman E, Freedman L, Rennert G, Paz-Elizur T, Livneh Z. Low integrated DNA repair score and lung cancer risk. Cancer Prev Res. 2014;7:398-406.

3. Sevilya Z, Leitner-Dagan Y, Pinchev M, Kremer R, Elinger D, Lejbkowicz F, Rennert HS, Freedman LS, Rennert G, Paz-Elizur T, Livneh Z. Development of APE1 enzymatic DNA repair assays: low APE1 activity is associated with increase lung cancer risk. Carcinogenesis. 2015;36(9):982-91. doi: 10.1093/carcin/bgv082. PubMed PMID: 26045303; PMCID: PMC4552243.

4. Leitner-Dagan Y, Sevilya Z, Pinchev M, Kramer R, Elinger D, Roisman LC, Rennert HS, Schechtman E, Freedman L, Rennert G, Livneh Z, Paz-Elizur T. N-Methylpurine DNA Glycosylase and OGG1 DNA Repair Activities: Opposite Associations With Lung Cancer Risk. J Natl Cancer Inst. 2012;104(22):1765-9. Epub 2012/10/30. doi: 10.1093/jnci/djs445. PubMed PMID: 23104324; PMCID: 3502197.

5. Love MI, Huber W, Anders S. Moderated estimation of fold change and dispersion for RNA-seq data with DESeq2. Genome Biol. 2014;15(12):550. Epub 2014/12/18. doi: 10.1186/s13059-014-0550-8. PubMed PMID: 25516281; PMCID: PMC4302049.

6. Subramanian A, Tamayo P, Mootha VK, Mukherjee S, Ebert BL, Gillette MA, Paulovich A, Pomeroy SL, Golub TR, Lander ES, Mesirov JP. Gene set enrichment analysis: a knowledge-based approach for interpreting genome-wide expression profiles. Proc Natl Acad Sci U S A. 2005;102(43):15545-50. Epub 2005/10/04. doi: 10.1073/pnas.0506580102. PubMed PMID: 16199517; PMCID: PMC1239896.

7. Team RC. R: A language and environment for statistical computing. R Foundation for Statistical Computing, Viena, Austria. 2015.

8. Wickham H. Ggplot2: Elegant graphics for data analysis. New York Springer-Verlag 2009.

9. Cassidy A, Myles JP, van Tongeren M, Page RD, Liloglou T, Duffy SW, Field JK. The LLP risk model: an individual risk prediction model for lung cancer. Br J Cancer. 2008;98(2):270-6. doi: 10.1038/sj.bjc.6604158; PMCID: PMC2361453.

Supplementary Tables

Supplementary Table 1. Variation of the DNA repair score with disease staging*

Lung cancer stage						T staging					
Stage	n	mean	STD	CI_lower	CI_upper	T	n	Mean	STD	CI lower	CI upper
C[†]	140	4.00	0.98	3.84	4.16	C[†]	140	4.00	0.98	3.84	4.16
1a	25	2.95	0.95	2.56	3.35	1a	22	2.80	1.02	2.35	3.25
1b	20	3.26	0.97	2.80	3.71	1b	17	2.65	1.050	2.11	3.19
2a	18	2.58	1.04	2.06	3.10	2a	49	2.84	1.03	2.54	3.13
2b	9	2.07	0.71	1.53	2.61	2b	19	2.45	1.05	1.94	2.95
3a	24	2.76	0.76	2.44	3.08	3	16	2.32	0.90	1.84	2.80
3b	8	2.18	0.86	1.46	2.90	4	24	2.62	1.32	2.06	3.15
4	44	2.44	1.28	2.06	2.83						

N staging						M staging					
N	n	mean	STD	CI_lower	CI_upper	M	n	Mean	STD	CI lower	CI upper
C[†]	140	4.00	0.98	3.84	4.16	C[†]	140	4.00	0.98	3.84	4.16
0	64	2.90	1.05	2.64	3.17	0	103	2.76	0.96	2.58	2.95
1	17	2.67	1.39	1.96	3.39	1a	14	2.37	1.22	1.67	3.07
2	51	2.50	0.95	2.23	2.77	1b	30	2.48	1.32	1.99	2.97
3	15	2.22	1.00	1.67	2.77						

		Cases vs. controls[‡]		Linear regression[§]			
		Difference	P value	Estimate	Std. Error	t value	Pr(>|t|)
Stage	Nonadjusted	-1.05	<0.0001	-0.103	0.038	-2.721	0.0073
	Adjusted	-0.79	0.0009	-0.125	0.039	-3.253	0.0014
T	Nonadjusted	-1.20	<0.0001	-0.065	0.055	-1.173	0.2426
	Adjusted	-0.67	0.011	-0.073	0.055	-1.329	0.1861
N	Nonadjusted	-1.09	<0.0001	-0.215	0.080	-2.700	0.0078
	Adjusted	-0.84	<0.0001	-0.252	0.081	-3.124	0.0022
M	Nonadjusted	-1.24	<0.0001	-0.160	0.108	-1.475	0.1425
	Adjusted	-1.05	<0.0001	-0.227	0.111	-2.047	0.0425

* DNA repair score with the revised APE1 assay.

[†] Control subjects

[‡] For disease stage - Controls vs stage 1a; for T staging - controls versus T1a; for N staging – controls vs. N0; for M staging, controls vs. M0.

[§] Test for a linear trend between staging categories in cases (control subjects not included).

Supplementary Table 2. Highest GSEA differentially expressed pathways in samples from lung cancer patients

No.	Pathway	Size*	ES†	NES‡	FDR q-val§
1	GO_POSITIVE_REGULATION_OF_IMMUNE_RESPONSE	450	-0.59	-2.75	$<10^{-5}$
2	GO_ACTIVATION_OF_IMMUNE_RESPONSE	340	-0.60	-2.73	$<10^{-5}$
3	GO_CYTOKINE_MEDIATED_SIGNALING_PATHWAY	344	-0.59	-2.72	$<10^{-5}$
4	GO_ACTIVATION_OF_INNATE_IMMUNE_RESPONSE	180	-0.62	-2.68	$<10^{-5}$
5	GO_REGULATION_OF_INNATE_IMMUNE_RESPONSE	300	-0.59	-2.67	$<10^{-5}$
6	GO_POSITIVE_REGULATION_OF_DEFENSE_RESPONSE	296	-0.58	-2.65	$<10^{-5}$
7	GO_POSITIVE_REGULATION_OF_INNATE_IMMUNE_RESPONSE	216	-0.60	-2.65	$<10^{-5}$
8	GO_ANTIGEN_PROCESSING_AND_PRESENTATION_OF_ EXOGENOUS_PEPTIDE_ANTIGEN_VIA_MHC_CLASS_I	60	-0.71	-2.65	$<10^{-5}$
9	GO_INFLAMMATORY_RESPONSE	336	-0.57	-2.64	$<10^{-5}$
10	GO_IMMUNE_RESPONSE_REGULATING_CELL_SURFACE_ RECEPTOR_SIGNALING_PATHWAY	256	-0.59	-2.63	$<10^{-5}$
11	GO_ANTIGEN_RECEPTOR_MEDIATED_SIGNALING_PATHWAY	153	-0.61	-2.62	$<10^{-5}$
12	GO_ANAPHASE_PROMOTING_COMPLEX_DEPENDENT_ CATABOLIC_PROCESS	72	-0.67	-2.60	$<10^{-5}$
13	GO_CELLULAR_RESPONSE_TO_CYTOKINE_STIMULUS	473	-0.55	-2.57	$<10^{-5}$
14	GO_GRANULOCYTE_MIGRATION	50	-0.71	-2.57	$<10^{-5}$
15	GO_RESPONSE_TO_INTERFERON_GAMMA	111	-0.63	-2.56	$<10^{-5}$
16	GO_INNATE_IMMUNE_RESPONSE	437	-0.55	-2.56	$<10^{-5}$
17	GO_LEUKOCYTE_CHEMOTAXIS	87	-0.64	-2.54	$<10^{-5}$
18	GO_CELL_CHEMOTAXIS	128	-0.60	-2.52	$<10^{-5}$
19	GO_INNATE_IMMUNE_RESPONSE_ACTIVATING_CELL_ SURFACE_RECEPTOR_SIGNALING_PATHWAY	94	-0.63	-2.52	$<10^{-5}$
20	GO_TUMOR_NECROSIS_FACTOR_MEDIATED_ SIGNALING_PATHWAY	99	-0.63	-2.52	$<10^{-5}$
21	GO_IMMUNE_EFFECTOR_PROCESS	374	-0.54	-2.51	$<10^{-5}$
22	GO_DEFENSE_RESPONSE_TO_BACTERIUM	124	-0.60	-2.51	$<10^{-5}$
23	GO_T_CELL_RECEPTOR_SIGNALING_PATHWAY	126	-0.61	-2.51	$<10^{-5}$
24	GO_ADAPTIVE_IMMUNE_RESPONSE	193	-0.57	-2.51	$<10^{-5}$
25	GO_LEUKOCYTE_MEDIATED_IMMUNITY	129	-0.60	-2.49	$<10^{-5}$
26	GO_REGULATION_OF_LEUKOCYTE_MEDIATED_IMMUNITY	131	-0.59	-2.49	$<10^{-5}$
27	GO_CELLULAR_RESPONSE_TO_INTERFERON_GAMMA	93	-0.62	-2.48	$<10^{-5}$
28	GO_POSITIVE_REGULATION_OF_IMMUNE_EFFECTOR_PROCESS	129	-0.59	-2.48	$<10^{-5}$
29	GO_MYELOID_LEUKOCYTE_MIGRATION	70	-0.64	-2.47	$<10^{-5}$
30	GO_PATTERN_RECOGNITION_RECEPTOR_SIGNALING_PATHWAY	96	-0.61	-2.45	$<10^{-5}$

*Number of genes in the pathway †Enrichment Score ‡Normalized enrichment score §False Discovery Rate q-value

Supplementary Table 3. Summary of biological pathways enrichment in nasal cells obtained for the DNA repair score using GSEA

Subjects class	Direction of correlation	Number of pathways reported by GSEA	Number of Biological pathways* with P value <0.001			
			All	Immune system*	Cell cycle*	Other
Case	Negative	3564	305	185†	23	97
Control	Negative	2638	92	1	40	51
Case	Positive	532	0	0	0	0
Control	Positive	1458	0	0	0	0

*Immune related pathways were selected based on the following keywords: IMMUNE, IMMUNITY, CHEMOTAXIS, CHEMOKINE, TUMOR_NECROSIS, B_CELL, T_CELL, LEUKOCYTE, GRANULOCYTE, LYMPHOCYTE, INTERFERON, ANTIGEN, DEFENSE, CYTOKINE, INFLAMM, MYELOID, FC_#RECEPTOR, MHC_#, KAPPAB, INTERLEUKIN, TOLL_LIKE_RECEPTOR, RESPONSE_TO_#VIRUS, MACROPHAGE, WOUND, PHAGO, NEUTROPHIL, RESPONSE_TO_#BACTERI, RESPONSE_TO_#FUNGUS; According to these keywords 366 out of the 4096 pathways reported by GSEA were defined as Immune related pathways.

Cell Cycle related pathways were selected based on the following keywords: CHROMATID, CENTROMERIC, DIVISION, SPINDLE, CHROMOSOM, KINETOCHORE, CELL_CYCLE, CENTROMERE, ANAPHASE, DNA_REPLICATION, MITOTIC, CYCLI. According to these keywords 123 out of the 4096 pathways reported by GSEA were defined as Cell Cycle related pathways.

The Symbol # is used to indicate the possible existence of several words with different endings.

Supplementary Table 4. Estimated projected 5-year risk of lung cancer for persons aged 65y with different smoking histories and different DNA repair scores

Gender	Duration of smoking	Average 5-year risk to develop lung cancer (%)*	5-year risk to develop lung cancer (%) for different DNA repair OMA scores[†]		
			≤5th percentile	≤10th percentile	≥75th percentile
Male	Never	0.4	1.7	1.3	0.1
	10y	0.8	3.6	2.8	0.2
	30y	1.6	6.9	5.4	0.4
	50y	4.5	17.2	13.8	1.1
Female	Never	0.2	1.1	0.9	0.1
	10y	0.5	2.4	1.8	0.1
	30y	1.1	4.6	3.6	0.2
	50y	3.0	12.0	9.5	0.7

* Average risk based on the Liverpool Lung Project Cancer Risk Model (Cassidy et al, Br. J. Cancer, 2008, 98(2):270-6).
[†]DNA repair risk values calculated assuming that the DNA repair score is normally distributed in the population, independent of age, sex and smoking history and there is no interaction between DNA repair score and these factors. See Methods section above.
Note that NLST participants had an approximate 5-year lung cancer risk of 3%. The table shows that, in the absence of DNA repair information, those aged 65y would have this risk or higher if they had smoked 50y. However, with information on the DNA repair score, it can be seen that those who have smoked 30y would also have an average risk of 3% or more if they have a low OMA score (below 10th percentile), whereas those who have smoked 50y but have a high OMA score (above 75th percentile) would have an average risk far below 3%.

Supplementary Figures

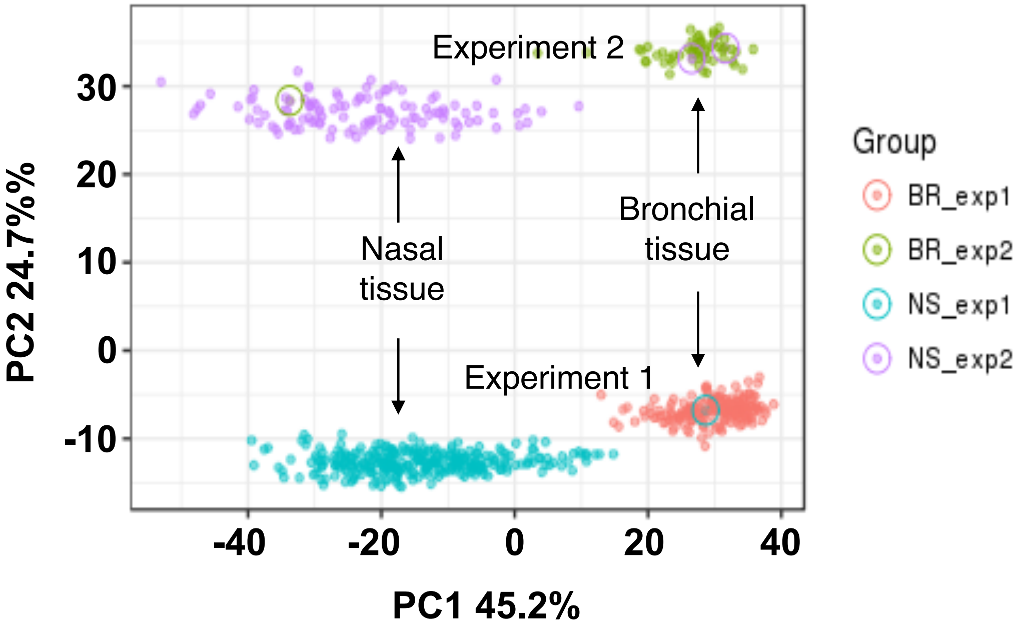

Supplementary Figure 1. Principle component analysis to detect the main sources of variation in the RNAseq data. NS, nasal samples; BR, bronchial samples; exp1, experiment 1, exp2, experiment 2.

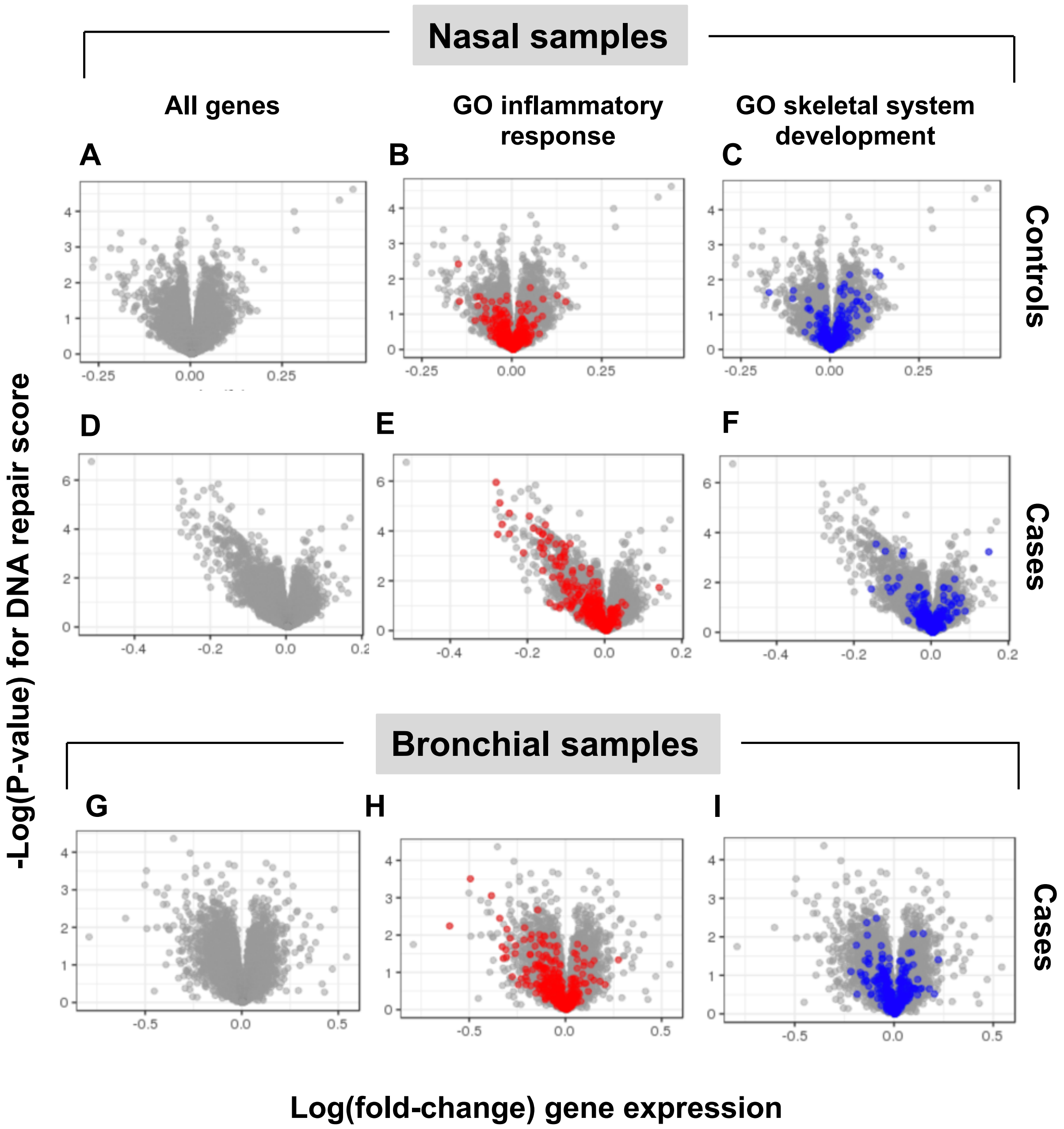

Supplementary Figure 2. Differential expression analysis relative to DNA repair OMA score for all available groups (Cases/Controls)x(Nasal/Bronchial) is presented by volcano plots. Grey dots represent non-annotated genes; Red dots represent genes from the inflammatory response pathway; Blue dote represents genes from the skeletal system development pathway.

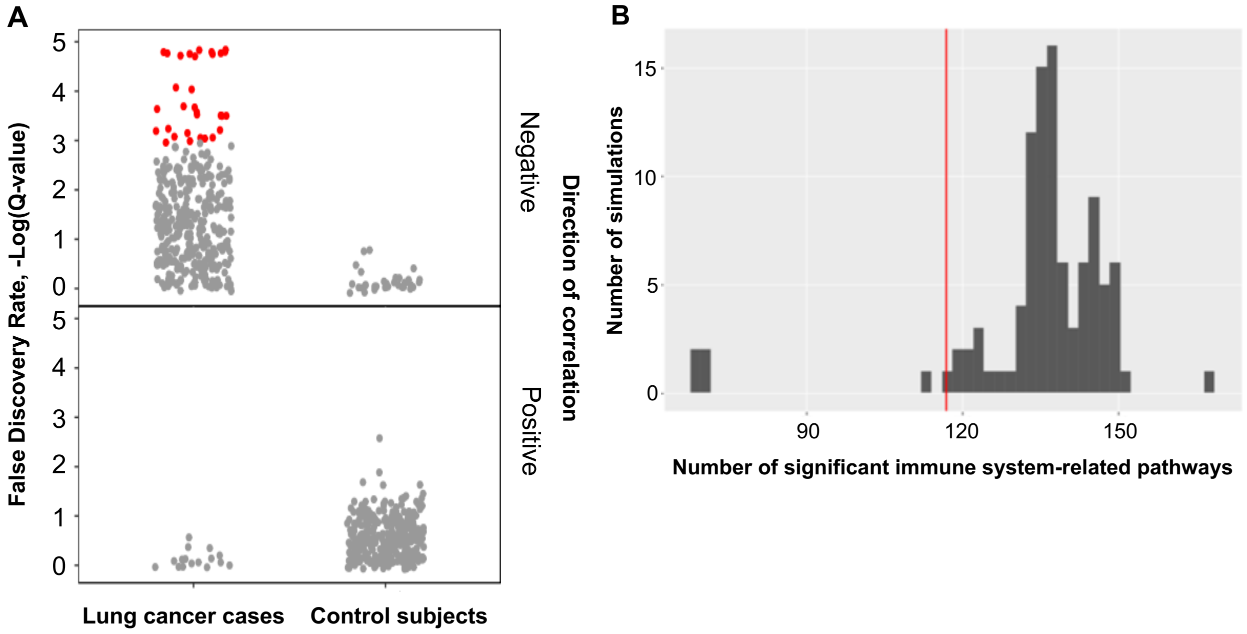

Supplementary Figure 3. Analysis of the robustness of the correlation between low DNA repair OMA score and upregulation of immune-system related pathways. A. Effect of extreme OMA score trimming on the enrichment of immune system pathways with low DNA repair score using gene set enrichment analysis (GSEA). Trimming was performed separately for nasal samples from cases and controls by removing samples with DNA repair OMA values in the 3.5% extreme OMA values from both sides. Genes from the trimmed sub-groups were ranked by the RNAseq2 analysis according to their correlation to the DNA repair score, and analyzed by GSEA to identify pathways (using GO terms) which significantly correlate (negative or positive correlation) with the DNA repair score. The figure represents all Immune system related pathways (depicted by the list of keywords presented in Supplementary Table 3) found in the GSEA analysis, with Y axis value showing the FDR -Log(Q-Value) for the enrichment score. The pathways were colored according to their FDR values: gray dots Q-Value > 0.001, red dots Q-Value<0.001. B. Sub-sampling analysis of the GSEA results. Analysis was performed on each of 100 simulations of random sub-sampling of 80% of the sample size. The graph presents the distribution, among the 100 simulations, of immune system-related pathways which had a stringent P-value of <0.001.

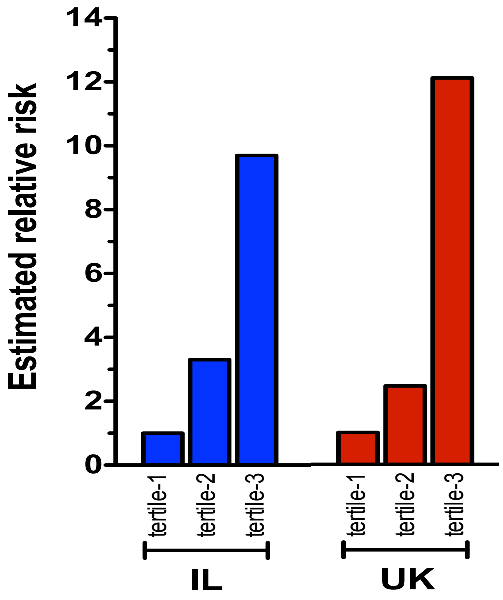

Supplementary Figure 4. Comparison of association of low DNA repair score with lung cancer in the UK and Israeli (IL) studies. Results of logistic regression, in which odds ratios were estimated for the continuous DNA repair score variable and categorized into 3 groups. IL, results taken from the Israeli study (2); UK, results were taken from Table 3 in the manuscript.
